# Association between inflammatory markers (SII and SIRI) and anxiety levels in Parkinson’s disease

**DOI:** 10.3389/fpsyt.2025.1635817

**Published:** 2025-09-02

**Authors:** Wen Zhou, Tianfang Zeng, Duan Liu, Ruijuan Pang, Liang Gong

**Affiliations:** Neurology Department, Chengdu Second People’s Hospital, Chengdu, Sichuan, China

**Keywords:** Parkinson’s disease, anxiety, systemic immune-inflammation index (SII), systemic inflammation response index (SIRI), inflammation, cross-sectional study

## Abstract

**Background:**

Parkinson’s disease (PD) is a progressive neurodegenerative disorder frequently associated with anxiety, which can significantly impair patients’ quality of life. Emerging evidence suggests that systemic inflammation may contribute to the development of anxiety in PD. The Systemic Immune-Inflammation Index (SII) and Systemic Inflammation Response Index (SIRI) are composite biomarkers reflecting systemic inflammatory status. However, the relationship between these inflammatory markers and anxiety levels in PD patients remains to be elucidated.

**Objective:**

To investigate the association between SII and SIRI and anxiety levels in PD patients.

**Methods:**

This cross-sectional study utilized data from the PPMI database, including 1,289 PD patients. Anxiety levels were assessed using the State-Trait Anxiety Inventory (STAI), with separate evaluations for state and trait anxiety. Linear regression analyses were performed to assess the associations between SII, SIRI, and anxiety levels. Curve fitting analysis was conducted to explore potential non-linear relationships, and sensitivity and subgroup analyses were performed to verify the robustness of the results.

**Results:**

Linear regression analyses showed significant positive associations between SII and overall STAI scores [β = 0.34 (95% CI 0.07 - 0.6), p = 0.014], STAI-state [β = 0.21 (95% CI 0.06 - 0.36), p = 0.005], and a non-significant association with STAI-trait [β = 0.13 (95% CI - 0.01 - 0.26), p = 0.073]. SIRI was significantly associated with overall STAI scores [β = 0.16 (95% CI 0.04 - 0.27), p = 0.008], STAI-state [β = 0.1 (95% CI 0.04 - 0.17), p = 0.002], and a non-significant association with STAI-trait [β = 0.06 (95% CI 0 - 0.12), p = 0.068]. Curve fitting analysis revealed no significant non-linear relationships between SII/SIRI and anxiety levels, indicating a linear positive correlation. Sensitivity and subgroup analyses confirmed the robustness of these findings.

**Conclusion:**

Our study demonstrates a significant positive linear association between SII and SIRI and anxiety levels, particularly state anxiety, in PD patients. These findings suggest that systemic inflammation may play a role in the development of anxiety in PD and highlight the potential utility of SII and SIRI as biomarkers for anxiety in this population. Future longitudinal studies are warranted to explore the causal relationship and potential therapeutic implications.

## 1 Introduction

Parkinson’s disease (PD) is a progressive neurodegenerative disorder characterized by motor symptoms such as tremor, rigidity, and bradykinesia. In addition to these motor manifestations, PD is frequently accompanied by a range of non-motor symptoms, among which anxiety is one of the most common neuropsychiatric complications. Anxiety in PD is characterized by persistent feelings of worry, difficulty concentrating, muscle tension, headaches, and insomnia (1). The prevalence of anxiety symptoms in PD patients is substantial, with approximately 31% of patients exhibiting anxiety symptoms (2), and up to 67% of PD patients being diagnosed with anxiety disorders (3). The high prevalence of anxiety and its significant impact on the quality of life of PD patients underscore the importance of early detection and identification of biomarkers and risk factors associated with anxiety in PD (4, 5).

Anxiety in PD patients is associated with worsening disease severity and a significant correlation with poorer quality of life compared to the general population (6). It is positively correlated with the severity of PD (7). Moreover, anxiety in PD has been linked to increased mortality (8). The presence of anxiety and depression in PD is associated with multiple adverse outcomes, including more severe motor symptoms, advanced disease stages, disability, and neuropsychiatric comorbidities such as cognitive impairment, sleep disturbances, and even autonomic dysfunction (9).

In recent years, there has been growing interest in the role of systemic inflammation in the pathogenesis of non-motor symptoms in PD. Two novel inflammatory markers, the Systemic Immune-Inflammation Index (SII) and the Systemic Inflammation Response Index (SIRI), which are assessed based on platelets and three types of white blood cell subtypes, have been proposed and have shown associations with cognitive function, depression and anxiety (10, 11). Previous research has revealed that inflammation and anti-inflammatory cytokines modulate anxiety through their respective receptors within the same basolateral amygdala (12). Various anti-inflammatory treatments have shown certain efficacy in unstratified anxiety patient populations (13, 14). However, the relationship between SII and SIRI and anxiety levels in PD patients remains to be fully elucidated.

Our study aims to investigate the association between SII and SIRI and anxiety levels in PD patients using data from the Parkinson’s Progression Marker Initiative (PPMI) database. Identifying this association could lead to early detection and inform intervention strategies, potentially enhancing patient outcomes. Moreover, understanding this relationship could provide crucial insights into the pathophysiology of anxiety in PD and may uncover new targets for therapeutic interventions aimed at managing anxiety in this patient population.

## 2 Methods and materials

### 2.1 Data source and ethical considerations

Data used in the preparation of this article was obtained on [2025-3-21] from the Parkinson’s Progression Markers Initiative (PPMI) database (www.ppmi-info.org/access-data-specimens/download-data), RRID: SCR_006431. For up-to-date information on the study, visit www.ppmi-info.org. The PPMI database is a publicly available resource that includes detailed clinical assessments and laboratory measurements from patients with PD. As this study utilized publicly available and de-identified data from the PPMI database, no additional ethical approval was required. The PPMI study was conducted in accordance with the Declaration of Helsinki, and all participants provided informed consent as part of the PPMI protocol.

### 2.2 Study population

**Figure 1:**
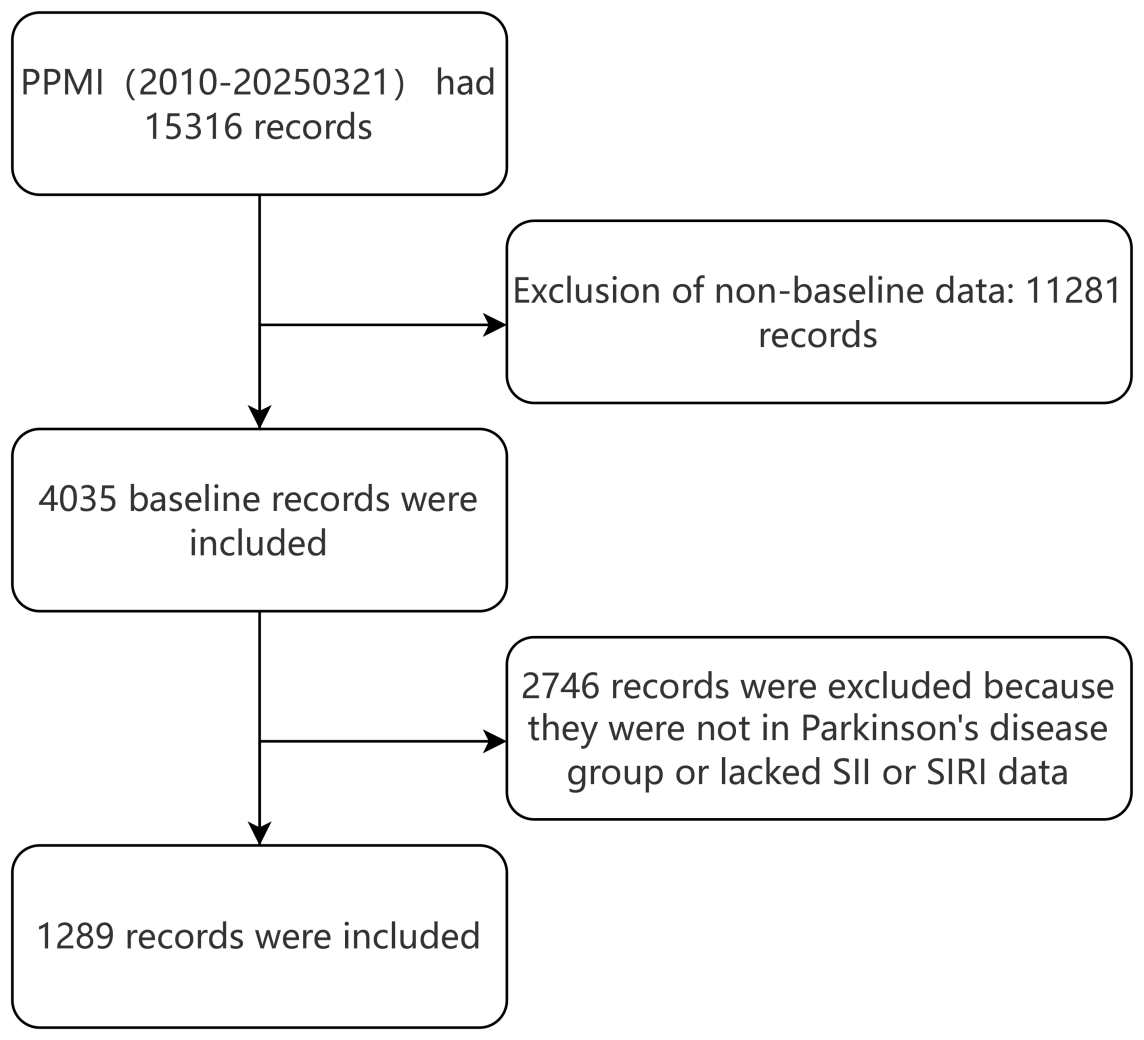
Flowchart of the study cohort.

The study population consisted of PD patients with complete data on anxiety levels, SII, and SIRI (**Figure 1**). Anxiety levels were assessed using the State-Trait Anxiety Inventory (STAI), which includes both state and trait anxiety subscales. Motor symptoms were evaluated using the Movement Disorder Society-Unified Parkinson’s Disease Rating Scale (MDS-UPDRS), a comprehensive tool that assesses both motor and non-motor symptoms in PD. Laboratory assessments of hematological and biochemical parameters were uniformly conducted at Covance laboratories.

### 2.3 Calculation of inflammatory markers

SII= Platelet count × Neutrophil count/Lymphocyte count.

SIRI= Neutrophil count × Monocyte count/Lymphocyte count.

### 2.4 Statistical analysis

Baseline characteristics were summarized using descriptive statistics. Normally distributed variables were reported as mean ± standard deviation (SD), while non-normally distributed measures were expressed as median (interquartile range, IQR). Categorical data were reported as frequencies and percentages.

To evaluate the association between the inflammatory markers (SII and SIRI) and anxiety levels in PD patients, we employed linear regression models using multiple imputation data. Three models were constructed to adjust for potential confounders based on clinical relevance, existing scientific literature, and their known associations with the outcomes of interest, including cases where they led to a change in the effect estimate exceeding 10%. Model 1 was unadjusted; Model 2 was adjusted for age, sex, body mass index (BMI), and education years; Model 3 was further adjusted for PD duration and MDS-UPDRS Part 3 score; Model 4 was further adjusted for Immunomodulatory drug usage, and SSRIs usage.

Curve fitting analysis was conducted to explore the potential non-linear relationships between SII/SIRI and anxiety levels. Specifically, we used restricted cubic splines to model the relationship between these variables. Sensitivity analysis was performed by excluding patients with any missing data to verify the robustness of the primary findings. Subgroup analyses were conducted to assess the consistency of the associations across different demographic and clinical characteristics. The subgroups included age (<65 years and ≥65 years), sex (male and female), BMI (<25 kg/m^2^, 25 – 30 kg/m^2^, and ≥30 kg/m^2^), PD duration (<3 years and ≥3 years), MDS-UPDRS Part 3 score (<33 and ≥33), PD age-onset group (Early-Onset PD <50 years old, Late-Onset PD ≥50 years old), and PD genetic group (Sporadic PD and Familial PD). Each subgroup analysis was performed using the same linear regression models as described above.

In this study, we aimed to investigate the role of various inflammatory and clinical indices in predicting anxiety status using machine learning algorithms. Anxiety status was defined based on the STAI-state score, with a cutoff value of 40 to determine the presence (STAI-state ≥ 40) or absence (STAI-state < 40) of anxiety symptoms. The dataset comprised features such as SIRI, SII, PD duration, age, sex, education years, race, BMI, UPDRS 3 score, STAI-trait score, and several blood cell ratios including Eosinophil-to-Lymphocyte Ratio (ELR), Neutrophil-to-Lymphocyte Ratio (NLR), Monocyte-to-Lymphocyte Ratio (MLR), Neutrophil-to-Platelet Ratio (NPR), Eosinophil-to-Neutrophil Ratio (ENR), and Platelet-to-Lymphocyte Ratio (PLR). Initially, we employed the Boruta algorithm for feature selection to identify key variables from this pool. Following the selection of significant features by Boruta, we constructed predictive models using these variables. To ensure robustness, models were trained with a phased integration framework involving human-provided data followed by machine processing for adaptive model architecture. We performed 5-fold cross-validation and utilized grid search for hyperparameter optimization. The optimal model was chosen based on the maximum Area Under the Receiver Operating Characteristic Curve (AUC). The Linear Discriminant Analysis (LDA) model emerged as the best-performing model, which was further subjected to hyperparameter tuning and 5-fold cross-validation to ensure optimal performance and reliability. The effectiveness of the LDA model was assessed by comparing AUC values and SHAP-beeswarm plots.

## 3 Results

### 3.1 Baseline characteristics of the study population

A total of 1,289 PD patients were included in the study. The demographic and clinical characteristics of the study population are summarized in **Table 1**. The mean age of the participants was 62.9 ± 9.7 years, with 62.2% being male. The mean age at onset of PD was 59.9 ± 10.3 years, and the median disease duration was 2.1 (IQR 1.2, 3.4) years. The mean MDS-UPDRS Part 3 score was 22.4 ± 10.1, indicating a range of motor symptom severity. Anxiety levels, as assessed by the State-Trait Anxiety Inventory (STAI), showed a mean total score of 65.6 ± 19.0, with mean state anxiety (STAI-state) and trait anxiety (STAI-trait) scores of 32.8 ± 10.3 and 32.8 ± 9.9, respectively. Laboratory assessments revealed a mean SII of 634.8 ± 375.6 and a median SIRI of 0.8 (IQR 0.6, 1.2).

**Table 1:** Demographic and clinical characteristics of the study population.

Variables	n (%)	Mean (SD)/Median (IQR)
Age(year), Mean ± SD		62.9 ± 9.7
Sex, n (%)		
Female	487 (37.8)	
Male	802 (62.2)	
Education years (year), Mean ± SD		15.8 ± 3.1
Race, n (%)		
White	1212 (94.5)	
Black	13 (1.0)	
Asian	16 (1.2)	
Other (includes multi-racial)	42 (3.3)	
BMI (kg/m^2^), Median (IQR)		26.2 (23.9, 29.6)
Onset age (years), Mean ± SD		59.9 ± 10.3
PD duration (years), Median (IQR)		2.1 (1.2, 3.4)
UPDRS 3 score, Mean ± SD		22.4 ± 10.1
HY, n (%)		
0	3 (0.2)	
1	424 (34.3)	
2	785 (63.5)	
3	25 (2.0)	
STAI, Mean ± SD		65.6 ± 19.0
STAI-state, Mean ± SD		32.8 ± 10.3
STAI-trait, Mean ± SD		32.8 ± 9.9
Lymphocytes, Mean ± SD		1.6 ± 0.5
Monocytes, Mean ± SD		0.4 ± 0.1
Neutrophils, Mean ± SD		3.9 ± 1.4
Platelets, Mean ± SD		242.8 ± 59.3
SSRIs use	239 (18.5)	
Immunomodulatory drug use	48 (3.7)	
SII, Mean ± SD		634.8 ± 375.6
SIRI, Median (IQR)		0.8 (0.6, 1.2)
NLR, Mean ± SD		2.6 ± 1.3
MLR, Mean ± SD		0.3 ± 0.1
LMR, Mean ± SD		4.6 ± 1.9
NAR, Mean ± SD		0.09 ± 0.03
PAR, Mean ± SD		5.6 ± 1.4
NPR, Mean ± SD		0.02 ± 0.01
ENR, Mean ± SD		0.04 ± 0.04
ELR, Mean ± SD		0.1 ± 0.08

N, number; BMI, body mass index; PD, Parkinson’s disease; race other, includes multi-racial; UPDRS, movement disorder society unified Parkinson’s disease rating scale; HY, Hoehn and Yahr Scale; STAI, state-trait anxiety inventory; STAI-state, state anxiety; STAI-trait, trait anxiety; SII, systemic immune inflammation index; SIRI, system inflammation response index; SSRIs, selective serotonin reuptake inhibitors; ELR, Eosinophil-to-Lymphocyte Ratio; NLR, Neutrophil-to-Lymphocyte Ratio; MLR, Monocyte-to-Lymphocyte Ratio; NPR, Neutrophil-to-Platelet Ratio; ENR, Eosinophil-to-Neutrophil Ratio; PLR, Platelet-to-Lymphocyte Ratio. Numbers that do not add up to 100% are attributable to missing data.

### 3.2 Association between inflammatory markers and anxiety levels

Linear regression analysis was conducted to assess the association between the inflammatory markers (SII and SIRI) and anxiety levels in PD patients, as measured by the STAI. The results are summarized in **Table 2**. For the total STAI score, a 100-unit increase in SII was associated with a 0.34-point increase in total STAI score (β = 0.34, 95% CI: 0.07 - 0.6, p = 0.014) after adjusting for age, sex, BMI, education years, PD duration, MDS-UPDRS Part 3 score, Immunomodulatory drug usage, and SSRIs usage (Model 4). Similarly, a 0.1-unit increase in SIRI was associated with a 0.16-point increase in total STAI score (β = 0.16, 95% CI: 0.04 - 0.27, p = 0.008) after adjusting for the same covariates. For the state anxiety subscale (STAI-state), a 100-unit increase in SII was associated with a 0.21-point increase in STAI-state score (β = 0.21, 95% CI: 0.06 - 0.36, p = 0.005) (Model 4). A 0.1-unit increase in SIRI was associated with a 0.1-point increase in STAI-state score (β = 0.1, 95% CI: 0.04 - 0.17, p = 0.002). For the trait anxiety subscale (STAI-trait), SII and SIRI did not show a significant association. These findings indicate that higher levels of SII and SIRI are significantly associated with increased state anxiety in PD patients, independent of potential confounders. The association with trait anxiety was not significant.

**Table 2:** Linear regression analysis of the association between inflammatory markers (SII and SIRI) and anxiety levels in PD.

	Variable	N total	Model 1		Model 2		Model 3		Model 4	
			β (95%CI)	P value	β (95%CI)	P value	β (95%CI)	P value	β (95%CI)	P value
STAI	SII	1289	0.35 (0.08~0.63)	0.012	0.38 (0.11~0.65)	0.006	0.35 (0.08~0.62)	0.011	0.34 (0.07~0.6)	0.014
	SIRI	1289	0.12 (0.01~0.24)	0.039	0.18 (0.06~0.3)	0.003	0.17 (0.05~0.29)	0.005	0.16 (0.04~0.27)	0.008
STAI state	SII	1289	0.22 (0.07~0.37)	0.004	0.23 (0.08~0.38)	0.002	0.22 (0.07~0.36)	0.004	0.21 (0.06~0.36)	0.005
	SIRI	1289	0.09 (0.02~0.15)	0.008	0.11 (0.05~0.18)	0.001	0.11 (0.04~0.17)	0.001	0.1 (0.04~0.17)	0.002
STAI trait	SII	1289	0.13 (-0.01~0.28)	0.068	0.15 (0.01~0.29)	0.038	0.14 (0~0.28)	0.058	0.13 (-0.01~0.26)	0.073
	SIRI	1289	0.04 (-0.02~0.1)	0.241	0.07 (0.01~0.13)	0.032	0.06 (0~0.12)	0.049	0.06 (0~0.12)	0.068

SII was entered as a continuous variable per 100-unit increase. SIRI was entered as a continuous variable per 0.1-unit increase.

STAI, state-trait anxiety inventory; STAI-state, state anxiety; STAI-trait, trait anxiety; SII, systemic immune inflammation index; SIRI, systemic inflammation response index; PD, Parkinson’s disease; n, number; BMI, body mass index; SSRIs, Selective Serotonin Reuptake Inhibitors.

Model 1: unadjusted.

Model 2: adjusted for age, sex, BMI, education years.

Model 3: adjusted for Model 2, duration of PD, MDS-UPDRS 3 score.

Model 4: adjusted for Model 3, Immunomodulatory drug usage, and SSRIs usage.

### 3.3 Curve fitting analysis

Our analysis of the potential non-linear relationships between SII/SIRI and anxiety levels, as measured by total STAI scores and STAI-state scores, revealed that a linear model sufficiently describes these associations (**Supplementary Figure 1**, all P for non-linearity ≥0.05).

### 3.4 Sensitivity analysis

To ensure the robustness of our findings, we conducted a sensitivity analysis by excluding patients with any missing data (**Table 3**). The results from this analysis were consistent with those from the primary analysis, thereby confirming the reliability of our findings.

**Table 3:** Sensitivity analysis of the association between inflammatory markers (SII and SIRI) and anxiety levels in PD.

	Variable	N total	Model 1		Model 2		Model 3		Model 4	
			β (95%CI)	P value	β (95%CI)	P value	β (95%CI)	P value	β (95%CI)	P value
STAI	SII	1209	0.34 (0.05~0.62)	0.02	0.36 (0.08~0.63)	0.013	0.34 (0.06~0.62)	0.017	0.32 (0.05~0.6)	0.021
	SIRI	1209	0.1 (-0.02~0.22)	0.096	0.15 (0.03~0.27)	0.012	0.15 (0.03~0.27)	0.015	0.14 (0.02~0.26)	0.021
STAI state	SII	1209	0.21 (0.05~0.36)	0.008	0.22 (0.06~0.37)	0.006	0.21 (0.05~0.36)	0.008	0.2 (0.05~0.35)	0.01
	SIRI	1209	0.07 (0.01~0.14)	0.028	0.1 (0.03~0.16)	0.004	0.09 (0.03~0.16)	0.005	0.09 (0.02~0.15)	0.007
STAI trait	SII	1209	0.13 (-0.02~0.28)	0.088	0.14 (-0.01~0.29)	0.059	0.13 (-0.01~0.28)	0.07	0.12 (-0.02~0.27)	0.086
	SIRI	1209	0.03 (-0.03~0.09)	0.367	0.06 (0~0.12)	0.071	0.06 (-0.01~0.12)	0.081	0.05 (-0.01~0.11)	0.109

SII was entered as a continuous variable per 100-unit increase. SIRI was entered as a continuous variable per 0.1-unit increase.

SII, systemic immune-inflammation index; SIRI, systemic inflammation response index; PD, Parkinson’s disease; n, number; BMI, body mass index; SSRIs, Selective Serotonin Reuptake Inhibitors.

Model 1: unadjusted.

Model 2: adjusted for age, sex, BMI, education years.

Model 3: adjusted for Model 2, duration of PD, MDS-UPDRS 3 score.

Model 4: adjusted for Model 3, Immunomodulatory drug usage, and SSRIs usage.

### 3.5 Subgroup analysis

**Figure 2:**
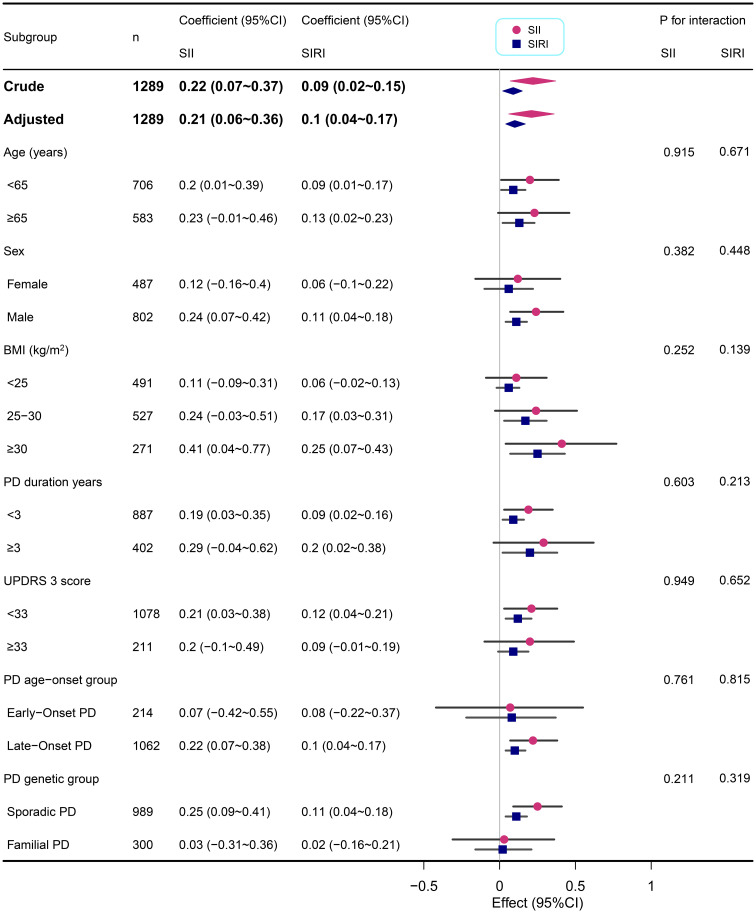
Subgroup analyses of the associations between inflammatory markers (SII and SIRI) and STAI-state in PD adjusted for age, sex, BMI, education years, duration of PD, MDS-UPDRS 3 score, Immunomodulatory drug usage, and SSRIs usage. In each case, the model was not adjusted for the stratification variable.

**Figure 3:**
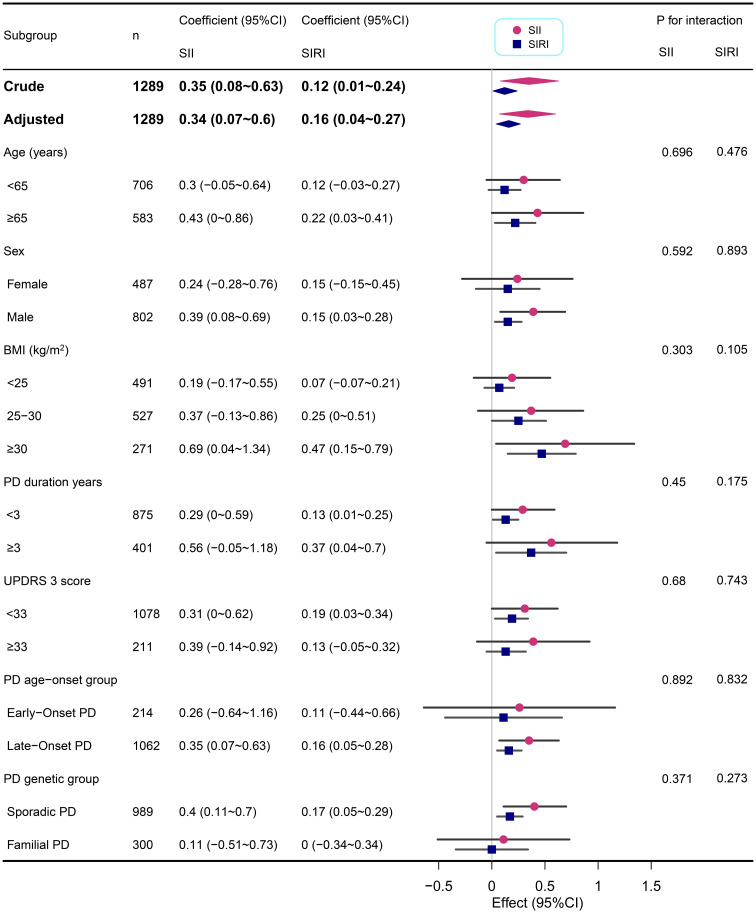
Subgroup analyses of the associations between inflammatory markers (SII and SIRI) and STAI in PD adjusted for age, sex, BMI, education years, duration of PD, MDS-UPDRS 3 score, Immunomodulatory drug usage, and SSRIs usage. In each case, the model was not adjusted for the stratification variable.

We performed subgroup analyses across various demographic and clinical characteristics, including age, sex, BMI, PD duration, MDS UPDRS 3 score, PD age-onset group, and PD genetic group (**Figures 2**, **3**). This suggests that the correlations between SII/SIRI and anxiety levels are both robust and largely unaffected by the characteristics examined in this analysis. Furthermore, the absence of significant interaction effects within these subgroups underscores the stability of these associations.

### 3.6 Machine learning performance

During the feature selection phase, we utilized the Boruta algorithm to screen all covariates. As illustrated in **Supplementary Figure 2**, the variables located in the green area, namely STAI-state, SII, SIRI, education years, PLR, NLR, MLR, ELR, ENR, and NPR, were identified as key factors for the model. These 10 feature variables were ultimately employed to construct the model. When selecting the most effective machine learning method, AUC serves as a pivotal criterion. Consequently, the LDA model was deemed optimal. The LDA model exhibited an average precision of 0.7765, an accuracy of 0.8498, a precision of 0.7765, and an F1 score of 0.673. The AUC values for both the training and test sets were 0.91, as shown in **Supplementary Figure 3**. It is worth noting that the SHAP-beeswarm plot highlighted the significant roles of SIRI and SII, as inflammatory indices, within the model (**Supplementary Figure 4**).

## 4 Discussion

Our study investigated the association between two novel inflammatory markers, SIRI and SII, and anxiety levels in PD patients. After comprehensive adjustment for potential confounders, we found that higher levels of SII and SIRI were significantly associated with increased state anxiety in PD patients. Specifically, a 100-unit increase in SII was associated with a 0.21-point increase in STAI-state score, and a 0.1-unit increase in SIRI was associated with a 0.1-point increase in STAI-state score. The association with trait anxiety was not significant. These findings highlight the potential role of systemic inflammation in the state of anxiety in PD patients.

Previous study has demonstrated increased SII levels are not only related to a higher risk of PD onset (HR = 1.04, 95% CI: 1.01 – 1.06, P = 0.013) but also to increased anxiety risk (HR = 1.03, 95% CI: 1.01 – 1.05, P = 0.025) (15). Logistics regression analysis indicated that higher SII was significantly correlated to the anxiety symptoms (*P* < 0.05) (11). Moreover, elevated neutrophil counts are significantly associated with increased anxiety risk (HR = 1.07, 95% CI: 1.04 – 1.10, P<0.001) (15). Additionally, higher lymphocyte counts or lymphocyte-to-monocyte ratios are associated with a reduced risk of PD (lymphocytes: HR = 0.73, 95% CI: 0.66 – 0.82, P<0.001; lymphocyte-to-monocyte ratios: HR = 0.78, 95% CI: 0.65 – 0.93, P = 0.013) (15). Low platelet counts are associated with an increased risk of PD onset (HR = 0.89, 95% CI: 0.84 – 0.95, P<0.001) (15). The impact of lower lymphocyte counts on PD risk may be causal (per 1-SD decrease, OR = 1.09, 95% CI: 1.01 – 1.18, P = 0.02) (16). These findings suggest that changes in inflammatory markers may play a significant role in the pathogenesis of PD. Moreover, studies on COVID-19 survivors have revealed a positive correlation between baseline SII and subsequent depression and anxiety scores (17). Elevated SII levels have also been associated with major depressive disorder (p = 0.002) (18), indicating that it may serve as a marker of low-grade inflammation observed in mood disorders (19). These findings underscore the broader relevance of inflammatory indices in neuropsychiatric conditions. Our study findings further support this notion, particularly in the context of anxiety, a non-motor symptom. We found that elevated inflammatory markers are closely correlated with state anxiety levels in PD patients.

The significant correlation between inflammatory indices and state anxiety in PD can be attributed to the complex interplay between peripheral and central inflammatory processes. Long-term activation of neutrophils can lead to tissue damage, as seen in various chronic inflammatory diseases (20–22). Neutrophils further amplify central inflammation by releasing granule proteins such as myeloperoxidase (23). These proteins trigger microglia, the brain’s resident immune cells, to adopt a pro-inflammatory M1 phenotype. M1 microglia secrete additional IL-6 and TNF-α (24). In PD, peripheral neutrophil activity is enhanced, while lymphocyte counts, particularly CD3+ and CD4+ T cells, are significantly reduced (25–27). This alteration may result from the migration of lymphocytes into the central nervous system, where they contribute to neuroinflammation. These infiltrating T cells also release pro-inflammatory cytokines such as TNF-α and IL-6, which directly damage dopaminergic neurons and activate microglia, establishing a positive feedback loop of neuroinflammation (24, 28).

Chronic neuroinflammation disrupts the blood-brain barrier (29), driven by matrix metalloproteinases and pro-inflammatory cytokines released by activated microglia (30). Increased blood-brain barrier permeability allows peripheral neutrophils to infiltrate the central nervous system, where they release reactive oxygen species and neurotoxic molecules, exacerbating oxidative stress and α-synuclein aggregation (29, 31).

Alterations in neuroanatomical circuits, such as the limbic cortex-striato-thalamocortical circuit (32) and the amygdala-insula pathway (33), further contribute to the development of anxiety in PD. The severity of anxiety has been reported to correlate with changes in the fear circuit (34). Prior literature has likewise demonstrated that T-cell subsets and neutrophils, by modulating inflammatory pathways, critically engage in maintaining the dynamic equilibrium of emotion-related neural networks (35, 36). Increased levels of cytokines, such as IL-6 and TNF-α, have been linked to anxiety-related brain regions including the prefrontal and limbic systems (37, 38). These cytokines can directly affect neuronal function and modulate the connectivity between the anterior cingulate cortex and amygdala, contributing to anxiety symptoms (39, 40). Systemic inflammation is a primary route that can lead to neuroinflammation, involving neural pathways, meningeal vessels, transport of cytokines across the blood-brain barrier, and secretion of cytokines by blood-brain barrier cells (41). The increased inflammation is associated with anxiety disorders and can be explained by the toxic effects of neuroinflammation on specific brain regions involved in each anxiety disorder (42). Future research could further explore these mechanistic pathways to clarify the precise role of inflammation in PD-related anxiety.

In this large, multi-center cross-sectional study, robust multivariable analyses, replicated across pre-specified subgroups and sensitivity checks, demonstrate that systemic immune-inflammation indices (SII and SIRI) are selectively associated with state, but not trait, anxiety in PD. These findings not only extend prior evidence for an inflammatory contribution to non-motor manifestations of PD but also highlight the potential clinical utility of SII and SIRI as biomarkers for state anxiety in PD patients. By identifying individuals at higher risk for state anxiety through elevated SII and SIRI scores, clinicians may be better positioned to implement timely surveillance and targeted intervention strategies, potentially improving patient outcomes.

Nevertheless, the design of this study precludes causal inference, residual confounding remains possible, and peripheral markers may imperfectly reflect central neuro-inflammation. These limitations, compounded by data-specific challenges such as a high attrition rate and minimal changes in anxiety scores over five years of follow-up, precluded a comprehensive longitudinal analysis in this study. Future research will address these limitations by incorporating longitudinal assessments, more detailed clinical records, neuroimaging, and cerebrospinal fluid biomarkers to better understand the progression of anxiety in PD, clarify the temporal dynamics of inflammation-driven anxiety, refine individual risk stratification, and inform precision enrollment in future therapeutic trials targeting neuropsychiatric symptoms in PD.

## 5 Conclusions

This study has revealed a significant association between inflammatory markers (SII and SIRI) and anxiety levels in patients with PD, pointing to inflammation’s possible role in PD-related anxiety. These results provide a foundation for future research exploring the complex relationship between inflammation and anxiety in PD and may inform the development of targeted intervention strategies.

## Data Availability

Publicly available datasets were analyzed in this study. This data can be found here: https://www.ppmi-info.org/access-data-specimens/download-datathe Parkinson’s Progression Markers Initiative (PPMI) zhouwen611@126.com.

## Supplementary material

Supplementary Figure 1 Linear dose response relationship between inflammatory markers (SII and SIRI) and anxiety levels in PD. Adjustment factors included age, sex, BMI, education years, duration of PD, MDS-UPDRS 3 score, Immunomodulatory drug usage, and SSRIs usage. The red line and red area represent the estimated values and their corresponding 95% confidence intervals, respectively.

Supplementary Figure 2 Boruta algorithm (B) are employed during the variable selection phase. The significance of potential predictors of anxiety status was assessed using the Boruta algorithm. The horizontal axis displays the names of the variables, while the vertical axis represents the Z-values for each variable. The box plots illustrate the Z-values during model calculations, with green boxes indicating important variables and red boxes denoting unimportant variables.

Supplementary Figure 3 The ROC of the machine learning models in train and test set.

Supplementary Figure 4 SHAP-beeswarm plot illustrating the contribution of each feature to the prediction of anxiety status. Each point represents a single observation from the dataset, positioned according to its SHAP value (horizontal axis) and colored by the value of the corresponding feature (vertical axis). Features are ranked vertically by their total impact on the model’s predictions, with higher values indicating greater influence. Positive SHAP values (right side of the plot) indicate features that contribute to a higher predicted probability of anxiety, while negative SHAP values (left side of the plot) indicate features that contribute to a lower predicted probability. The color gradient reflects the actual value of the feature, with higher values shown in red and lower values in blue.
