# Supplementary figures and images for: Association between inflammatory markers (SII and SIRI) and anxiety levels in Parkinson’s disease
Source: Front Psychiatry. 2025 Sep 2;16:1635817. doi: 10.3389/fpsyt.2025.1635817 (PMC12436417; doi:10.3389/fpsyt.2025.1635817)

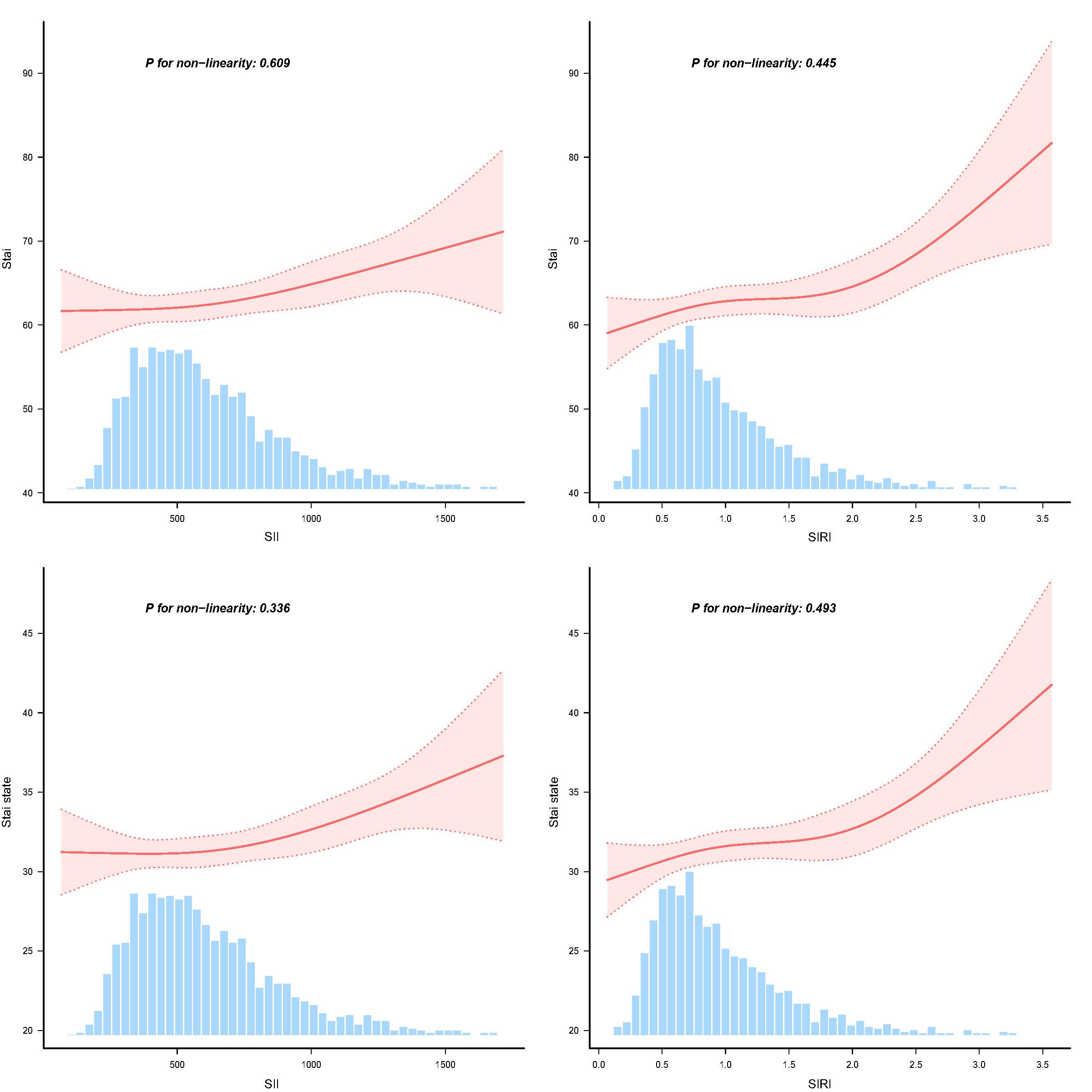

Supplement: Supplementary Figure 1 — Linear dose response relationship between inflammatory markers (SII and SIRI) and anxiety levels in PD. Adjustment factors included age, sex, BMI, education years, duration of PD, MDS-UPDRS 3 score, Immunomodulatory drug usage, and SSRIs usage. The red line and red area represent the estimated values and their corresponding 95% confidence intervals, respectively. [file Image1.jpeg]

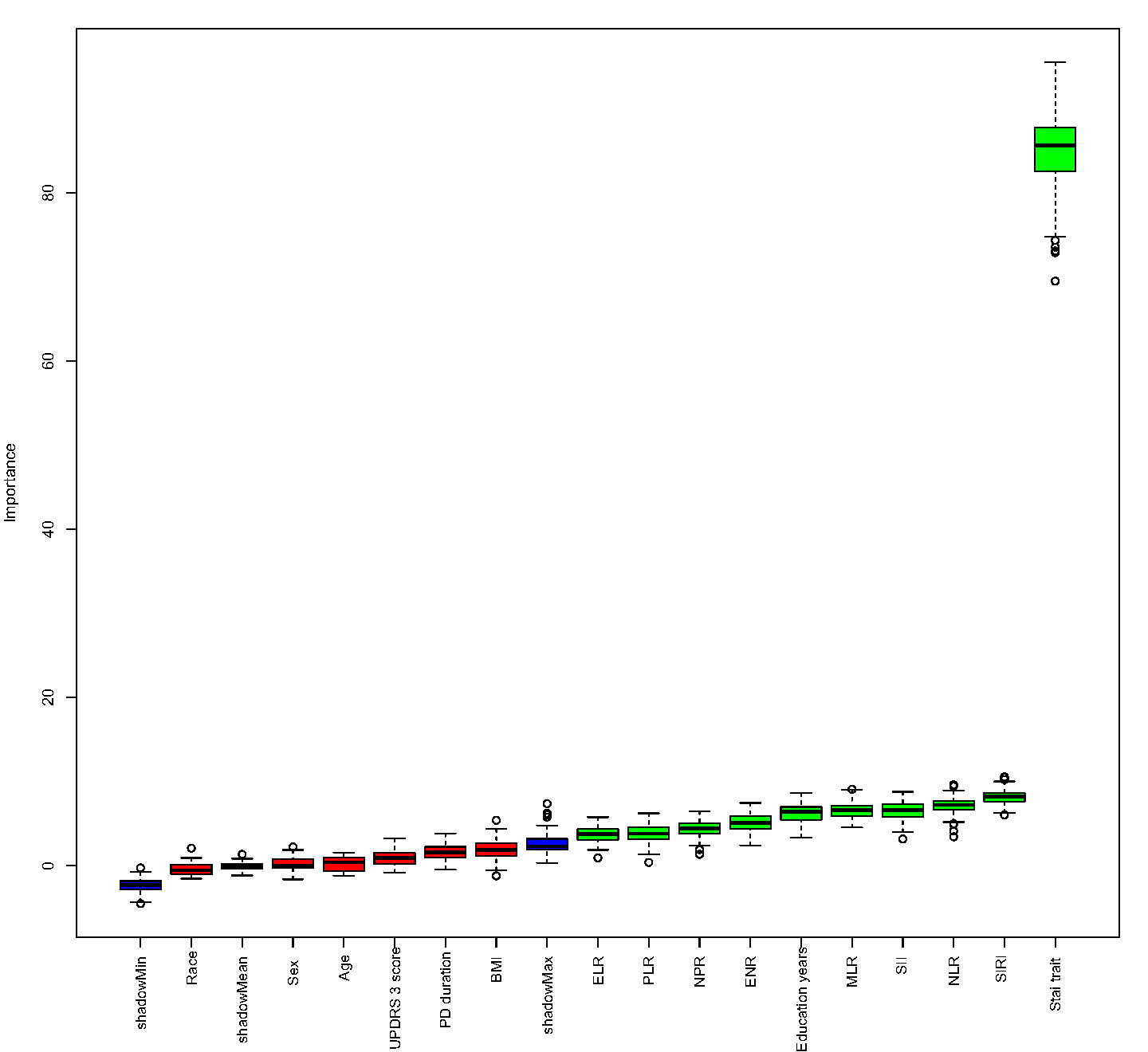

Supplement: Supplementary Figure 2 — Boruta algorithm (B) are employed during the variable selection phase. The significance of potential predictors of anxiety status was assessed using the Boruta algorithm. The horizontal axis displays the names of the variables, while the vertical axis represents the Z-values for each variable. The box plots illustrate the Z-values during model calculations, with green boxes indicating important variables and red boxes denoting unimportant variables. [file Image2.jpeg]

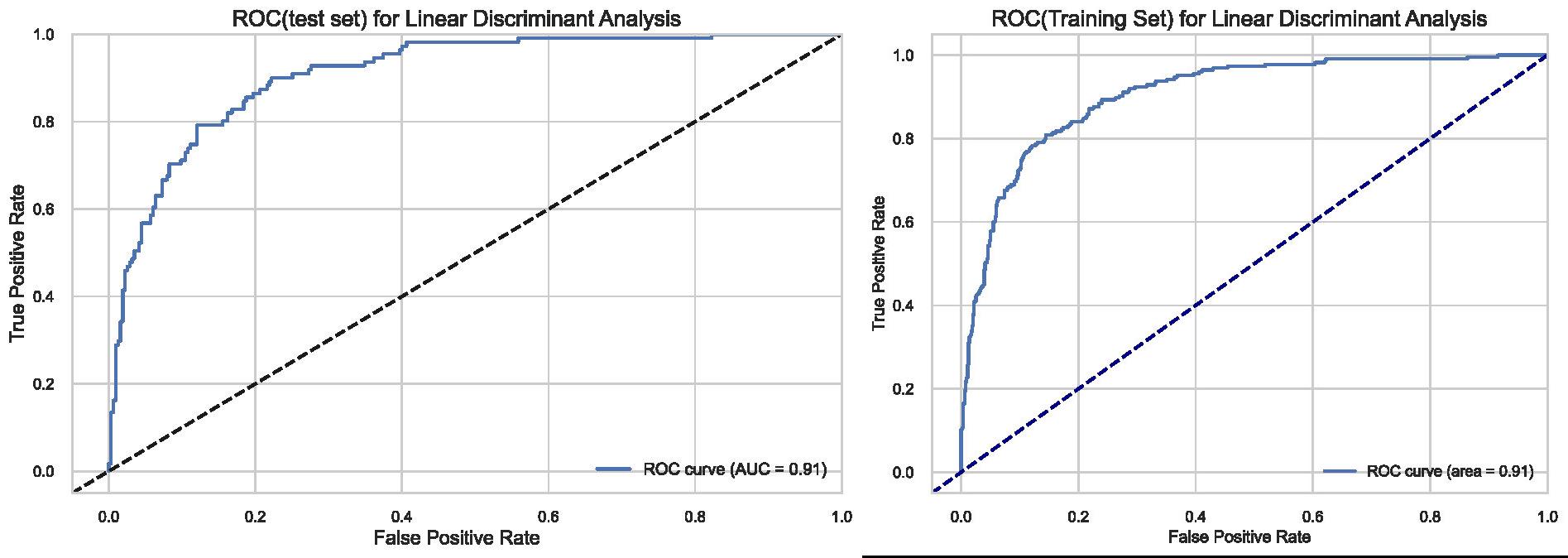

Supplement: Supplementary Figure 3 — The ROC of the machine learning models in train and test set. [file Image3.jpeg]

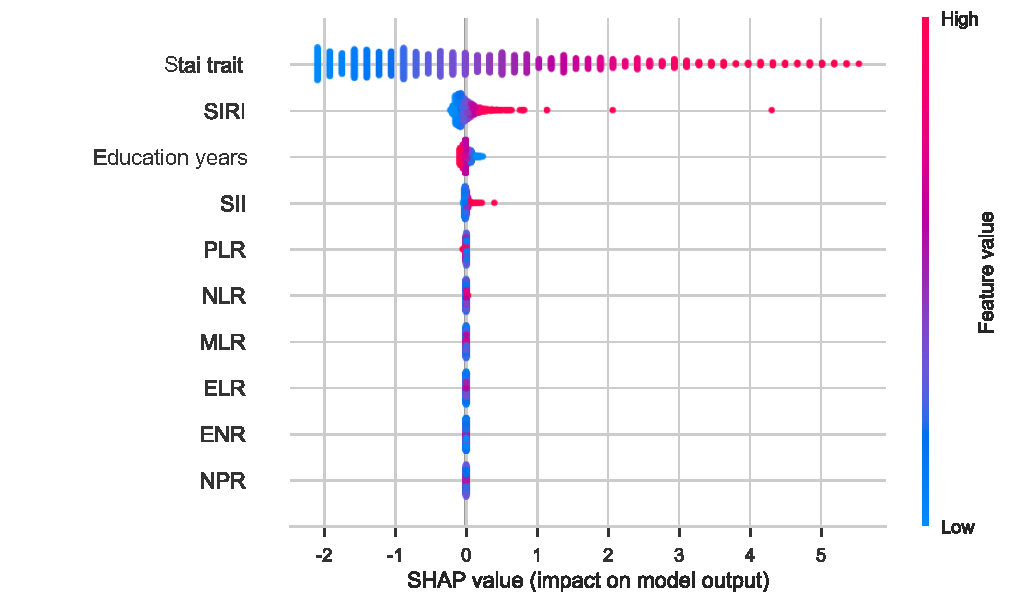

Supplement: Supplementary Figure 4 — SHAP-beeswarm plot illustrating the contribution of each feature to the prediction of anxiety status. Each point represents a single observation from the dataset, positioned according to its SHAP value (horizontal axis) and colored by the value of the corresponding feature (vertical axis). Features are ranked vertically by their total impact on the model’s predictions, with higher values indicating greater influence. Positive SHAP values (right side of the plot) indicate features that contribute to a higher predicted probability of anxiety, while negative SHAP values (left side of the plot) indicate features that contribute to a lower predicted probability. The color gradient reflects the actual value of the feature, with higher values shown in red and lower values in blue. [file Image4.jpeg]
